# Alleviating effect of vine tea on *Aeromonas hydrophila* infection revealed by small RNA transcriptome analyses of loach liver

**DOI:** 10.3389/fimmu.2025.1584985

**Published:** 2025-05-21

**Authors:** Wenqing Lai, Yuqin Qiu, Shengnan Pan, Aijun Wan, Xueyang Wang, Dingding Lv, Lishang Dai

**Affiliations:** ^1^ Jiangsu Key Laboratory of Sericultural Biology and Biotechnology, School of Biotechnology, Jiangsu University of Science and Technology, Zhenjiang, China; ^2^ The Key Laboratory of Silkworm and Mulberry Genetic Improvement, Ministry of Agriculture, Sericultural Research Institute, Chinese Academy of Agricultural Science, Zhenjiang, China; ^3^ School of Pharmaceutical Sciences, Wenzhou Medical University, Wenzhou, China; ^4^ Nursing School, Zhenjiang College, Zhenjiang, China

**Keywords:** microRNA, *Ampelopsis grossedentata*, loach, pathogen, innate immune

## Abstract

Vine tea (*Ampelopsis grossedentata*) is a widely used Chinese herb with a long history of application in southern China, known for its notable anti-infective, antibacterial and immunomodulatory pharmacological properties. It has potential for application in aquaculture as an inexpensive and readily available dietary supplement, promoting growth, antioxidant activity, and immune regulation in fish. However, there have been very few studies investigating the effects of vine tea on fish miRNAs. Loach is an economically important freshwater fish species, highly valued for its delicious flavor, but research on its miRNA relatively limited. To examine immune-related miRNAs in loach and to further determine the antibacterial immune mechanisms of vine tea, we performed small RNA sequencing analysis of loach liver tissue before and after treatment with vine tea extract. The results showed that vine tea could affect the antibacterial immune activity of loach through the miRNA regulation. A total of 25 differentially expressed miRNAs were identified in liver tissues, and KEGG pathway analysis revealed that most of these miRNAs were involved in innate immune responses such as autophagy, lysosomes, endocytosis, and pattern receptor signaling pathways. To the best of our knowledge, this is the first study to profile miRNA expression in loach after treatment with vine tea extract. This work deepens the understanding of the role of loach miRNAs in the immune system and opens new prospects for application of vine tea in aquaculture.

## 1 Introduction

MicroRNAs (miRNAs) are ~22-nucleotide highly conserved endogenous RNAs that govern gene expression post-transcriptionally across diverse living organisms (1, 2). Single-stranded miRNAs are often completely or incompletely complementarily paired with the 3’ untranslated region of the target mRNA, which leads to miRNA degradation or blocks the translation process of miRNA (3). The first miRNA (Lin-4) was discovered in *Caenorhabditis elegans* in 1993 (4), since then thousands of miRNAs have been extensively studied in animals, plants and viruses. Previous studies have shown that miRNAs regulate of a variety of biological processes *in vivo*, including development tissue, signal transduction, proliferation, apoptosis, differentiation, immune response, inflammation, and others (5–7). Given their importance, a large number of miRNAs have been identified by high-throughput sequencing and bioinformatics in fish, such as zebrafish (*Danio rerio*), grass carp (*Ctenopharyngodon idellus*), yellow croaker (*Pseudosciaena crocea*), tilapia (*Oreochromis niloticus*), mandarin fish (*Siniperca chuatsi*), and Blunt snout bream (*Megalobrama amblycephala*), etc. (8). Multiple studies show that miRNAs play crucial roles in fish physiology, nutrition, immune response, and immune escape (9). For example, miR-146a (10), miR1338-5p (11), miR-122 (12), and miR-181b-5p (13) regulate long-chain fatty acid synthesis and are involved in cholesterol metabolism, glycolipid metabolism, and musculogenesis, etc.; miR-146a (14), miR-3570 (15), miR-192 (16), miR-200a-3p (17)and miR-210 (18) play roles in antiviral and antibacterial immunity as well as promoting the immune response in fish. However, the miRNA network regulating fish immunity in aquaculture remains poorly understood, requiring further functional and mechanistic studies.

The loach (*Misgurnus anguillicaudatus*) is a Cobitidae family in the Cypriniformes order and is widely distributed throughout eastern Asia, including China, Japan, Korea and other countries (19, 20). Known for its high protein and low-fat contents, it is an excellent freshwater aquaculture species favored by consumers (21). Beyond its economic and nutritional value, it also serves as an important experimental animal model in research on growth performance, immune function, and other aspects. During culture, factors, including water quality, temperature and stocking density suppress immune function of loach, increasing their susceptibility to proto-microbial infections, specifically from Gram-negative bacteria. *Aeromonas hydrophila*, a Gram-negative, rod-shaped, highly virulent bacterium (22), is commonly occur in freshwater aquaculture and can cause ulcers, ascites, septicaemia, erythema, and liver damage in a wide range of fish species (23–25), leading to high mortality rates and significant economic losses, challenges that also influence the loach aquaculture industry.

In aquaculture, antibiotics are commonly used treat bacterial infections, however, their misuse not only leads to the emergence of drug resistance in pathogenic bacteria, but also threatens the ecological environment and public health (26, 27). In addition, aquaculture is often challenged by immunosuppression due to high-density farming, environmental stressors, and frequent pathogen exposure, leading to higher disease susceptibility and mortality rates. These challenges have created an urgent need for natural dietary supplements that can strengthen aquaculture immunity and reduce dependence on antibiotics (28). There are several lines of evidence that phytobiotics may be the future trend for antibiotic replacement in aquaculture, and they contain bioactive compounds with immune-enhancing and bactericidal properties (29). However, studies on plant-derived drugs related to antibacterial infection of loach and miRNA of loach were scare.

Vine tea (*Ampelopsis grossedentata*) is a precious traditional Chinese herb used as both medicine and food. It is a vine plant in the *Ampelopsis* genus of the Vitaceae family, widely distributed in southern regions (30–32). Vine tea has gained increasing attention due to its rich content, particularly its leaves, which are known as the ‘king of flavonoids’ they contain dihydromyricetin, providing various health benefits such as hypolipidemic, hypotensive, anti-inflammatory, antioxidant, antimicrobial, and immune boosting effects making it a popular health tea drink (33–35). Recent pharmacological research has shown that dihydromyricetin improves immune function via modulating cytokine production, lowering oxidative stress, and altering inflammatory pathways, including NF-κB signaling pathway (36, 37). This is because, Chinese government allowed to use its leaves as a feed ingredient for poultry and livestock, thereby improving the health of the organism and increasing the level of nutrition (38). In aquaculture and farm animals, Supplementation with vine tea extracts is a promising technique for improving fish health by increasing innate and adaptive immune responses, antioxidant defenses, and gut microbiome balance. Such properties suggest that vine tea could be used as functional feed additive to promote more sustainable and resilient aquaculture practices (39). For example, a previous study shown that the addition of moderate amounts of vine tea extracts to feeds promotes growth, induce antioxidant activity, and boost immunity in fish, supporting their healthy culture (40). However, further research is required to confirm the potential therapeutic role of vine tea in protecting fish against bacterial infection.

MiRNAs have been widely studied in fish nutrition and immune regulation (41), but no studies have been published on the influence of vine tea on miRNA expression profiles in fish. In light of this, we pre-added vine tea extract to the feed and investigated the dynamics of the miRNA transcriptome in the liver of loach following *A. hydrophila* infection. This study will, for the first time, demonstrate moderating effect of vine tea on fish after bacterial infection using comparative miRNA analysis, and investigate the immunomodulatory mechanism of vine tea. Our results contribute to better understanding of the role of miRNAs in loach and highlight the potential of vine tea extract as a feed additive for aquaculture.

## 2 Materials and methods

### 2.1 Feed and bacteria preparation

The vine tea extract was dissolved, and the solution was sprayed on the feed to achieve a final concentration of 0.01 kg vine tea/kg. The feed was air-dried and sealed and stored at 4°C. *A. hydrophila* was the strain available in the laboratory. *A. hydrophila* at -80°C was taken and added to fresh medium, recovered overnight and the concentration was determined, before the bacterial solution was stored at 4°C for subsequent use.

### 2.2 Animal trial and sample collection

The loaches for this experiment were obtained from a river fresh market in Wenzhou City, Zhejiang Province, China. The loach was transported to an aquarium and reared at room temperature of 25°C for 12 h light/dark cycle. Feed regular feed twice a day for a few days. After one week of domestication, 40 healthy loach samples (mean weight 10 ± 0.5 g) were randomly divided into two groups. The treatment group (AG+) was fed with 2.5 g of mixed feed with vine tea extract at a final concentration of 1 mg/ml, while the control group (AG-) group was fed with equal amount of normal feed. *A. hydrophila* suspension was added to the treatment and control groups, respectively, after 3 hours to maintain the bacterial concentration in the water column at 1 × 10^5^ CFU/ml. Each treatment group included 20 loaches to ensure sufficient biological replication. Twenty-four hours after bacterial infestation, three loaches from each of the treatment and control groups were selected for execution, and liver tissues were collected and stored at -80°C after liquid nitrogen flash freezing until total RNA was extracted for small RNA sequencing. All experimental procedures were conducted following standardized protocols to ensure consistency and reproducibility.

### 2.3 Construction and sequencing of two small RNA libraries

Total RNA was extracted from liver tissues of the treatment and control groups using Trizol reagent (Japan, TAKARA) according to the manufacturer’s instructions. RNA integrity was assessed by RNA Integrity Number (RIN) values in an Agilent 2100 Bioanalyzer (Agilent Technologies Inc, California, USA). RNA concentration and purity of were determined by measuring OD260 and OD260/OD280 using a NanoDrop 2000 Spectrophotometer (Thermo Scientific, Waltham, Massachusetts, USA). Libraries were prepared following the procedure of the NEB Next Multiplex Small RNA Library Prep Set for Illumina kit (New England Biolabs Inc, USA). Briefly, RNA were ligated to 5’ and 3’ sequencing junctions using ligase, then reverse transcribed into cDNA using RT primers. The cDNA was subsequently amplified by PCR with the adaptor specific primers. The purified PCR products were separated by 15% PAGE gel electrophoresis to construct small RNA library. Libraries concentrations were detected using an Agilent 2100 Bioanalyzer, and the quality of the libraries was checked using an Agilent High Sensitivity DNA Kit (Agilent Technologies Inc, USA). Finally, Single-End mode sequencing was performed on an Illumina sequencer, following the procedure of Personal Biotechnology Company Limited (China, Shanghai).

### 2.4 Sequencing reads analysis

Raw reads generated by high-throughput sequencing were filtered using an internally developed script to remove reads containing splices and low quality reads (average sequencing quality < 20). In addition, reads containing poly - N were removed, resulting in clean data. The number of clean reads with sequence lengths between 18 nt and 36 nt was then counted for each of the two libraries. After de-duplication of identical sequences within individual samples, the unique reads were blasted against the Rfam database (http://sanger.ac.uk/pub/databases/Rfam/) to remove four known types of ncRNAs, including rRNA, tRNA, snRNA and snoRNA. Reads that were not annotated as the above ncRNAs were subsequently aligned to mature miRNA sequences from all animals listed in miRBase database 22.0 (http://www.mirbase.org/) to identify conserved miRNAs, allowing up to two mismatch.

### 2.5 Differential analysis of miRNAs

Based on the number of sequences mapped to conserved miRNAs, the reads counts of statistically measured miRNAs were used to represent miRNA expression levels in each sample. To identify differentially expressed miRNAs between treatment and control groups, miRNAs counts were standardized. Differential expression analysis was performed using DESeq software, with the criterion for differential expression defined as an absolute log2 fold change (treatment group/control group) greater than 1. In addition, a P-value < 0.05 considered statistically significant.

### 2.6 Prediction and enrichment analysis of target genes

To further investigate the potential function of significantly differentially expressed miRNAs after vine tea extract treatment, target gene prediction was performed using miranda software. The 3’UTR sequences of homologous species genes were used as custom targets, 25 differentially expressed miRNAs were used as custom miRNAs. The miRNA threshold was set at a free energy of less than -20 kcal/mol. Enrichment analysis of the predicted target genes was performed with Gene Ontology (GO). First, all the predicted target genes were mapped to each GO term, the number of target genes in each term was counted, and P-value (the criterion for significant enrichment is P-value<0.05) were calculated by the hypergeometric distribution method, with the whole genome as the background. GO terms with P-values < 0.05 were considered significantly enriched. In addition, Kyoto Encyclopedia of Genes and Genomes (KEGG) enrichment analysis was conducted to better understand the biological functions of the target genes and to identify significantly enriched pathways.

### 2.7 Validation of differentially expressed miRNAs

To validate the differentially expressed miRNAs identified by high-throughput sequencing, six miRNAs were randomly selected for real-time fluorescence quantitative PCR (RT-qPCR) analysis. Forward primers based on mature miRNA sequence were designed by Primer5 software, and a universal reverse primer was used. Details of the forward primers and corresponding mature miRNAs are shown in **Table 1**. RNA reverse transcription was performed using the same total RNA samples as those used for library construction, following the instructions of the miRNA 1st Strand cDNA Synthesis Kit (by slicing A) (Vazyme, Nanjing, China). Subsequently, the qPCR reaction mixture was prepared according to the instructions of the TransStart^®^ Top Green qPCR SuperMix (+Dyei) (TransGen, Beijing) kit, including 1μL template cDNA, 0.5 μL of each primer (10 μmol·L-1), 10uL Top Green qPCR SuperMix and 8uL of RNase-free water. Finally, the reaction program was set up on a QuantStudio™ Real-Time PCR System (ABI, USA): 94°C for 30 s, 94°C for 5 s, 55°C for 15 s, 72°C for 10 s, and 40 cycles. The relative expression levels of miRNAs were assessed by the 2^-ΔΔCt^ method, and β-actin gene was used as the internal control for normalization. All experiments for each miRNA were performed in triplicate.

**Table 1:** Primers sequence for RT-qPCR.

miRNA	Forward primers (5’→3’)
manu-mir-194-3	CCAGTGGAGATGCTGTTACCTG
manu-undef-1012	CTGCATCAGGAACTGATTGGA
manu-undef-834	TTCCCTTTGTCATCCTATGCCTG
manu-undef-882	TAGCTTATCAGACTGGTGTTGGC
manu-mir-19-2	TGTGCAAATCCATGCAAAACTCG
manu-mir-130-16	CTTTGACGATGTTGCACTACT
β-actin	AGAGAGAAATTGTCCGTGAC

## 3 Results

### 3.1 Summary of miRNA sequencing data

**Figure 1:**
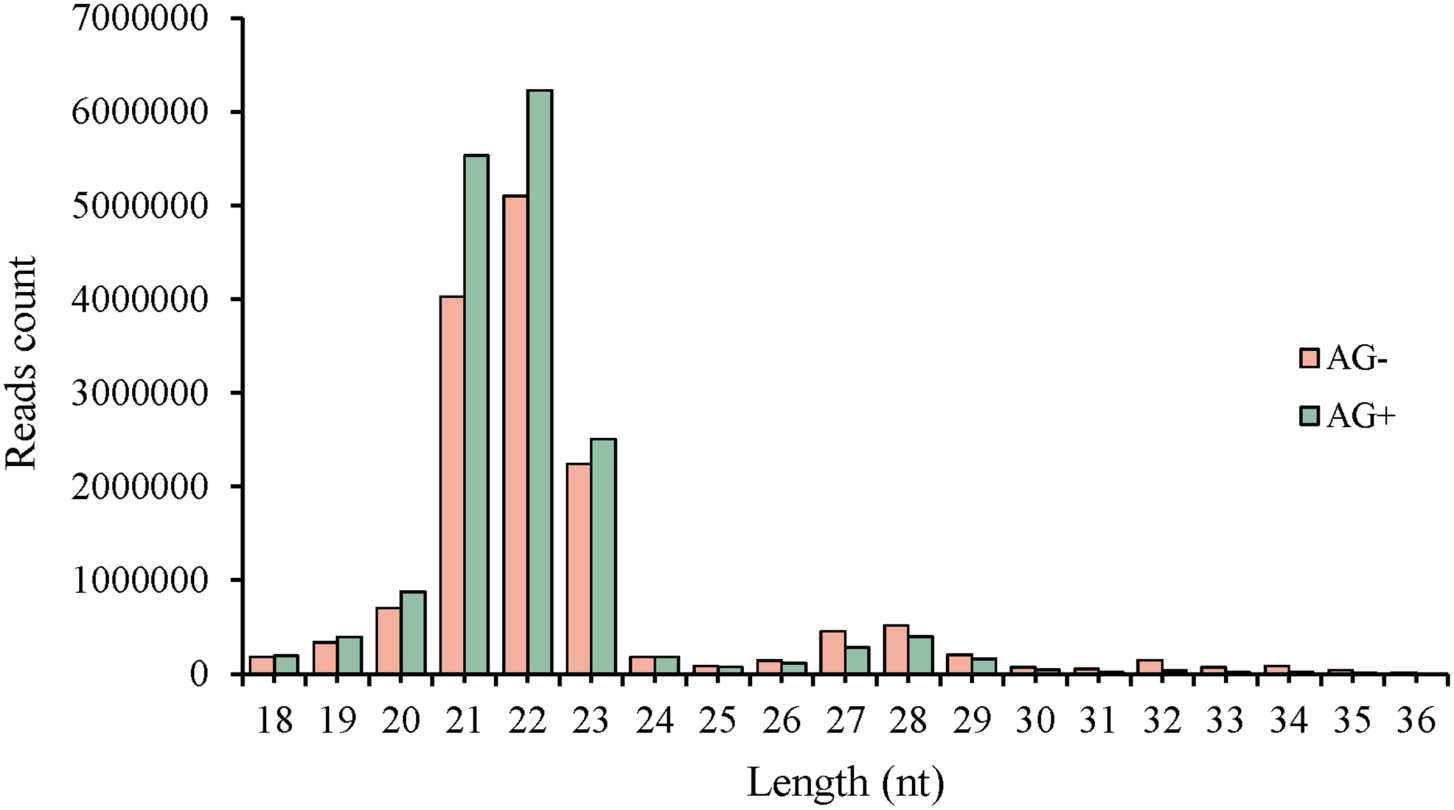
Length distribution of clean sequences from small RNA libraries in loach liver.

Liver tissues of pre-treated with vine tea extract before *A. hydrophila* (AG+) and tissues directly infected without pre-treatment (AG-) were used as samples for miRNA library construction and deep sequencing on Illumina platform. A summary of the two libraries is presented in **Table 2**. Sequencing generated 16,239,217 and 19,036,596 raw reads from the AG- and AG+ liver samples, respectively. After filtering of raw reads, including adaptor removal and mass clipping, a total of 14,660,513 and 17,102,503 clean reads (≥ 18 nt in length) were obtained for subsequent analysis. After removing duplicate identical sequences within each libraries, 350,436 and 343,509 unique reads were identified, respectively. The length distribution of the clean reads in the two libraries is shown in **Figure 1**, with the majority of reads distributed between 20–23 nt consistent with known size range of functional s RNAs (usually 20 ~ 24 nt) (42).

**Table 2:** Summary of preliminary analysis of sequencing data of two small RNA libraries.

Category	AG-	AG+
Raw reads	16239217	19036596
Clean reads	14660513	17102503
Clean reads%	90.28%	89.84%
Rfam database	2250829	1609923
miRBase database	11621891	14650679
Annotated%	94.63%	95.08%
Unique small RNAs	109066	92816
Mature miRNAs	924	982

Clean reads%: The percentage of clean reads count in raw reads count; Annotated%: The percentage of annotated reads count in Rfam and miRbase databases in total clean reads count; Unique small RNAs: Sequence count after deduplication of reads annotated in Rfam and miRbase databases; Mature miRNAs: The mature miRNA counts on the comparison in the miRBase database.

**Figure 2:**
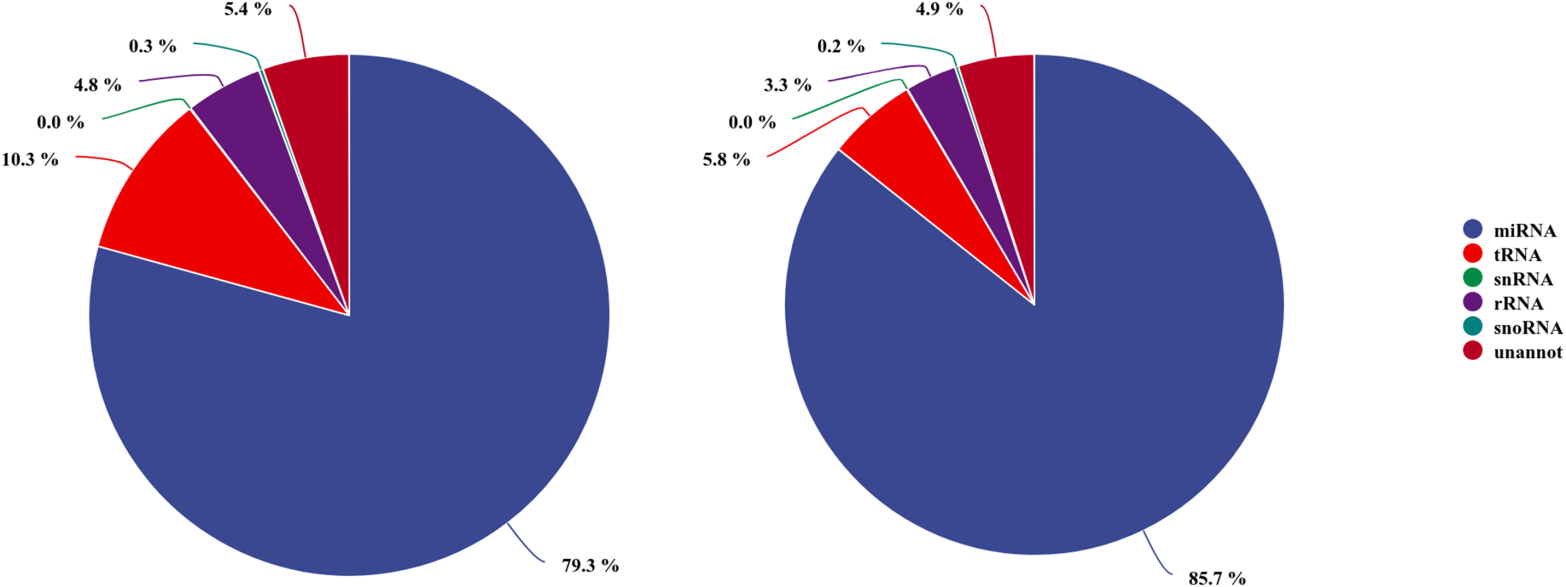
Annotation of small RNAs derived from AG-(left) and AG+(right) libraries.

The read length distribution were broadly similar between the two libraries, with 22nt reads showing the highest abundance, accounting for 34.80% and 36.41% of the clean reads in the AG– and AG+ libraries, respectively, followed by reads 21, 23 and 20nt in length. The clean reads were then mapped to the Rfam and miRBase databases, resulting in 13,872,720 and 16,260,602 annotated reads, corresponding to 94.63% and 95.08% of the clean reads from the AG- and AG+ libraries, respectively (**Figure 2**). These results indicated that most reads were successfully annotated to non-coding RNAs (rRNA, tRNA, snRNA, and snoRNA) or miRNAs, confirming the high quality of the sequencing data. In addition, the number of unique reads annotated to the small RNA database was 109,066 and 92,816 for the AG– and AG+ libraries respectively.

### 3.2 Differential expression of miRNAs

**Figure 3:**
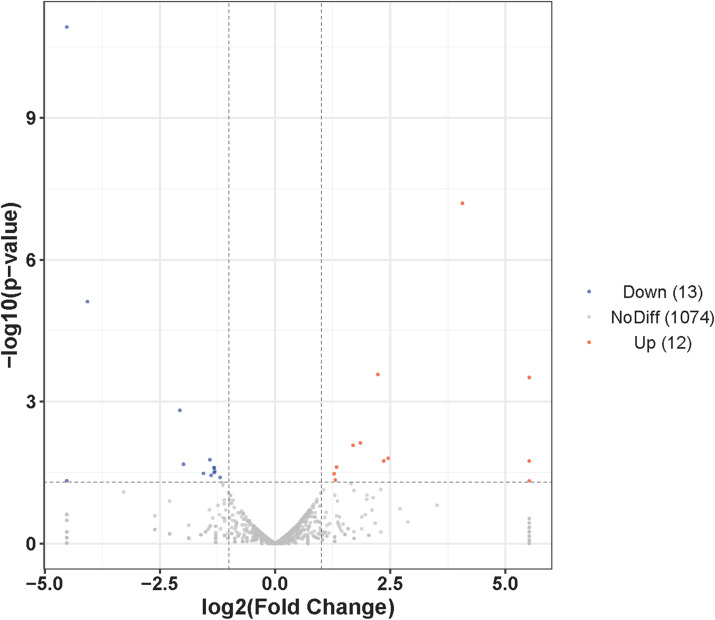
Volcano plot of the differentially expressed miRNAs in loach liver.

To identify conserved miRNAs in the AG- and AG+ libraries, data from both libraries were compared with mature miRNAs from all animals in the miRBase database. The results demonstrated that 924 and 982 conserved miRNAs were screened in the AG- and AG+, respectively, with 807 miRNAs were shared between the two libraries (**Table 2**, **Additional File 1**). Examination of the expression profiles of these miRNAs revealed that read counts varied widely, ranging from 0 to millions, indicating substantial differences in expression levels among different miRNAs. For example, manu-undef-553 exhibited the highest expression in both samples, with an average of 6,650,403 reads, followed by manu-mir-122-3 (1,603,475 reads) and manu-undef-668 (576,365 reads) (**Additional File 2**). To explore the anti-infection mechanisms in loach after vine tea pretreatment, it was crucial to screen differentially expressed miRNAs. In this study, analysis of miRNA expression levels between the AG- and AG+ groups revealed that 25 miRNAs were significantly differentially expressed (**Figure 3**). Among the 12 up-regulated miRNAs, t 2 were specific to the AG+ group, while among the 13 down-regulated miRNAs, 1 was specific to the AG- (**Table 3**). The number of reads for these differentially expressed miRNAs ranged from 0-24837, which is much lower than the expression of the highly abundant miRNAs mentioned above; they may play an important regulatory role in the immune response to *A. hydrophila* infection.

**Table 3:** The differentially expressed genes of loach after pretreatment with vine tea extract.

MiRNA	Reads count (AG-)	Reads count (AG+)	Fold Change	P-value
manu-mir-194-3	11.05541597	60.60378026	5.481818182	0.015736757
manu-mir-31-1	459.9053043	2161.836341	4.700611888	0.000267079
manu-undef-1012	451.0609715	1097.199783	2.432486631	0.03360853
manu-undef-286	0	14.47254454	Inf	0.047216667
manu-undef-385	130.4539084	322.91865	2.475346687	0.045340069
manu-undef-464	0	18.99521471	Inf	0.018072566
manu-undef-508	12.16095756	62.41284833	5.132231405	0.017938367
manu-undef-704	70.75466219	255.0785975	3.605113636	0.007453856
manu-undef-801	1.105541597	50.65390589	45.81818182	0.00030831
manu-undef-834	144.8259492	468.5486295	3.235253296	0.008385506
manu-undef-879	24.32191513	407.9448492	16.77272727	6.37678E-08
manu-undef-882	901.0164014	2275.807629	2.525822644	0.024293618
manu-mir-130-1	1186.246133	479.4030379	0.404134542	0.029905926
manu-mir-130-16	284.1241904	108.544084	0.382030421	0.036308882
manu-mir-19-2	2179.022487	867.4481384	0.398090494	0.025171935
manu-mir-203-1	466.5385538	111.2576861	0.238474795	0.001527916
manu-undef-401	8069.348115	350.9592051	0.043492882	1.19218E-11
manu-undef-412	137.087158	8.140806304	0.059384164	7.67808E-06
manu-undef-569	13.26649916	0	0	0.04704574
manu-undef-612	86.23224455	21.70881681	0.251748252	0.021107863
manu-undef-796	699.8078308	279.5010164	0.399396812	0.031463892
manu-undef-881	156.9869067	53.36750799	0.339948784	0.032978577
manu-undef-892	3493.511446	1400.218684	0.400805524	0.025229679
manu-undef-97	2833.503113	1062.82749	0.375093108	0.016956825
manu-undef-980	24837.09751	10852.59934	0.436951191	0.040143182

### 3.3 Validation of differentially expressed miRNAs by RT-qPCR

**Figure 4:**
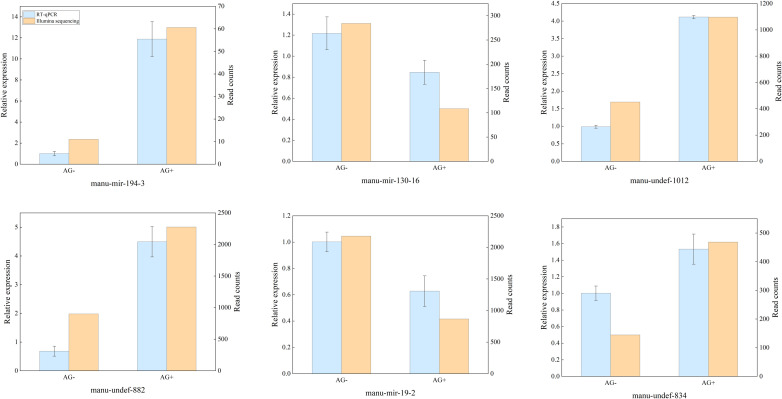
Comparison of relative expression levels between high-throughput sequencing and RT-qPCR results.

RT - qPCR was used to validate the differentially expressed miRNAs identified by high-throughput sequencing. Six immune-related miRNAs were selected for validation, include four up-regulated miRNAs (manu-mir-194-3, manu-undef-1012, manu-undef-834 and manu-undef-882) and two down-regulated miRNAs (manu-mir-19–2 and manu-mir-130-16). The results revealed that the expression patterns of these six miRNAs in the qRT-PCR assay were consistent with those observed in the high-throughput sequencing analyses (**Figure 4**). Although the relative expression levels used differed between the two methods, the trends in miRNAs expression were largely similar. These findings further confirmed the reliability and accuracy of the high-throughput sequencing data.

### 3.4 Targets prediction and functional analysis of miRNAs

The identification of target genes is essential for elucidating miRNA function. In this study, target prediction for the 25 differentially expressed miRNAs in loach liver was performed using miRanda software. Thousands of putative target genes were obtained through non-reference genome comparison, and multiple target sites were found on some target genes. The complexity of miRNA-mRNA interaction networks suggests that individual miRNAs can simultaneously regulate multiple target miRNAs, and that single mRNAs can, in turn, be regulated by multiple miRNAs. To infer the roles these differentially expressed miRNAs in the host, functional annotation and pathway enrichment analyses were performed on the predicted target genes.

**Figure 5:**
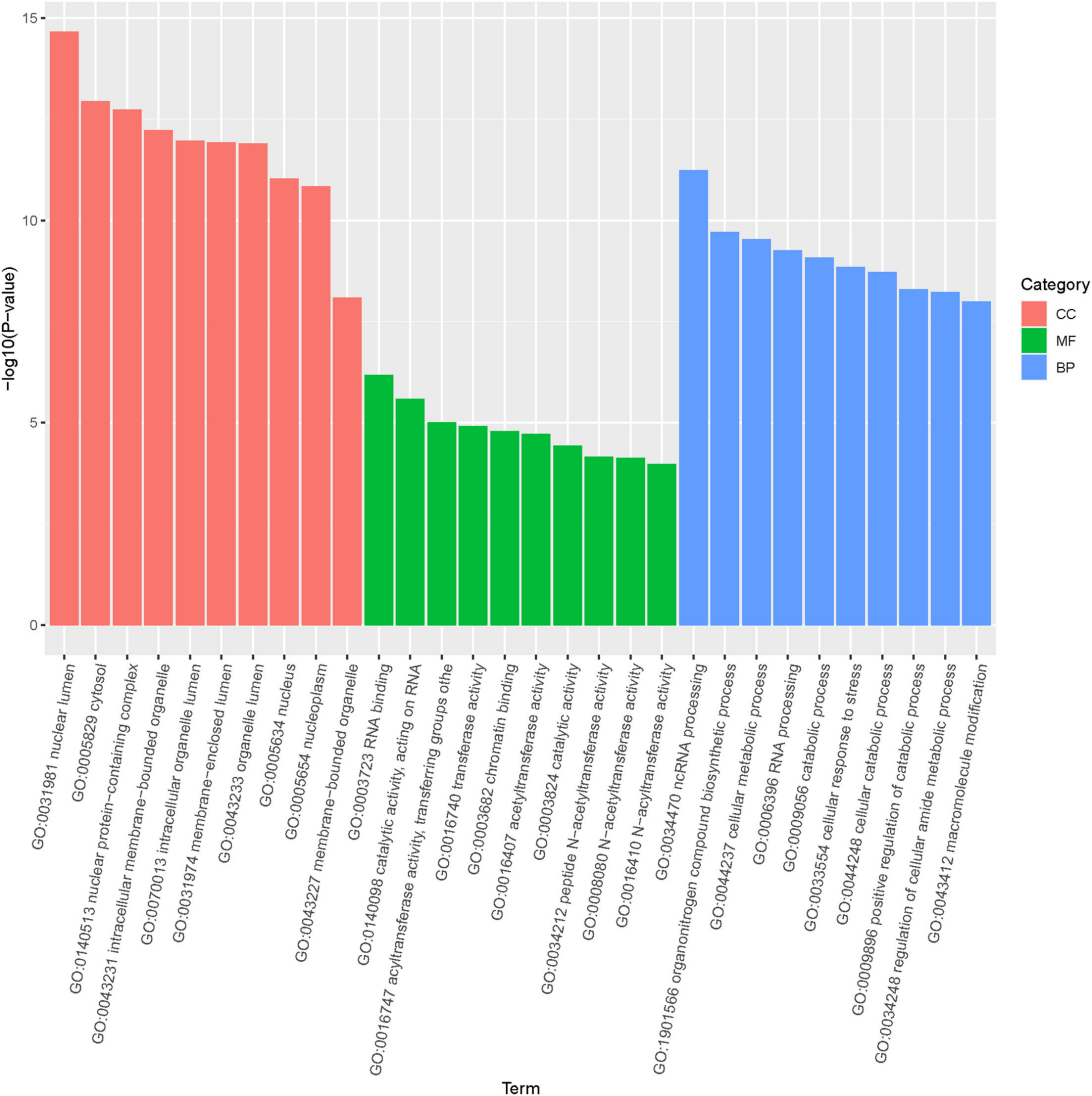
Gene ontology (GO) distribution of target genes of differentially expressed miRNAs in loach.

GO enrichment and KEGG pathway analysis were performed on the predicted target genes to identify the functions of the differentially expressed miRNAs. As shown in **Figure 5**, the GO annotations for target genes were distributed across three main categories: molecular function, biological process and cellular component. In terms of cell components, the predicted target genes were mainly clustered in the nucleus, organelles, and cytoplasm. For biological processes, the most enriched terms include catabolic process, biosynthetic process and cellular stress response, with cellular stress being closely related to immune response. Regarding molecular functions, the target genes were primarily involved in translocation, binding, and catalytic activities.

**Figure 6:**
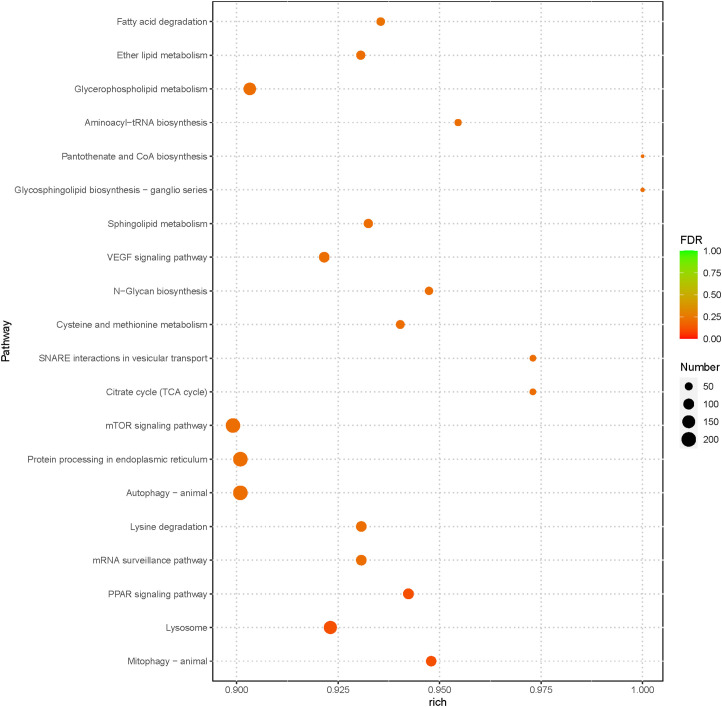
The 20 most enriched KEGG pathways enriched by the putative target genes of the differentially expressed miRNAs from AG- and AG+ samples.

Furthermore, to identify the pathways regulated by miRNAs during bacterial infection in loach, KEGG pathway enrichment of the target genes was conducted using clusterProfiler software. The results showed a total of 160 enriched pathways, with the ‘MAPK signaling pathway’ showing the highest number of target genes (378 genes) (**Additional File 3**). Among the top 20 significantly enriched pathways, the target genes were mainly involved in lipid metabolism, amino acid metabolism, carbohydrate metabolism, and signal transduction-related pathways (**Figure 6**). Noteably, immune system-related pathways such as lysosomes, autophagy, and protein processing in the endoplasmic reticulum were also significantly enriched, implying that the majority of the differentially expressed miRNAs may exert their anti-infective effects by modulating immune-related pathways.

### 3.5 Immune-related signaling pathways enriched for target genes

**Figure 7:**
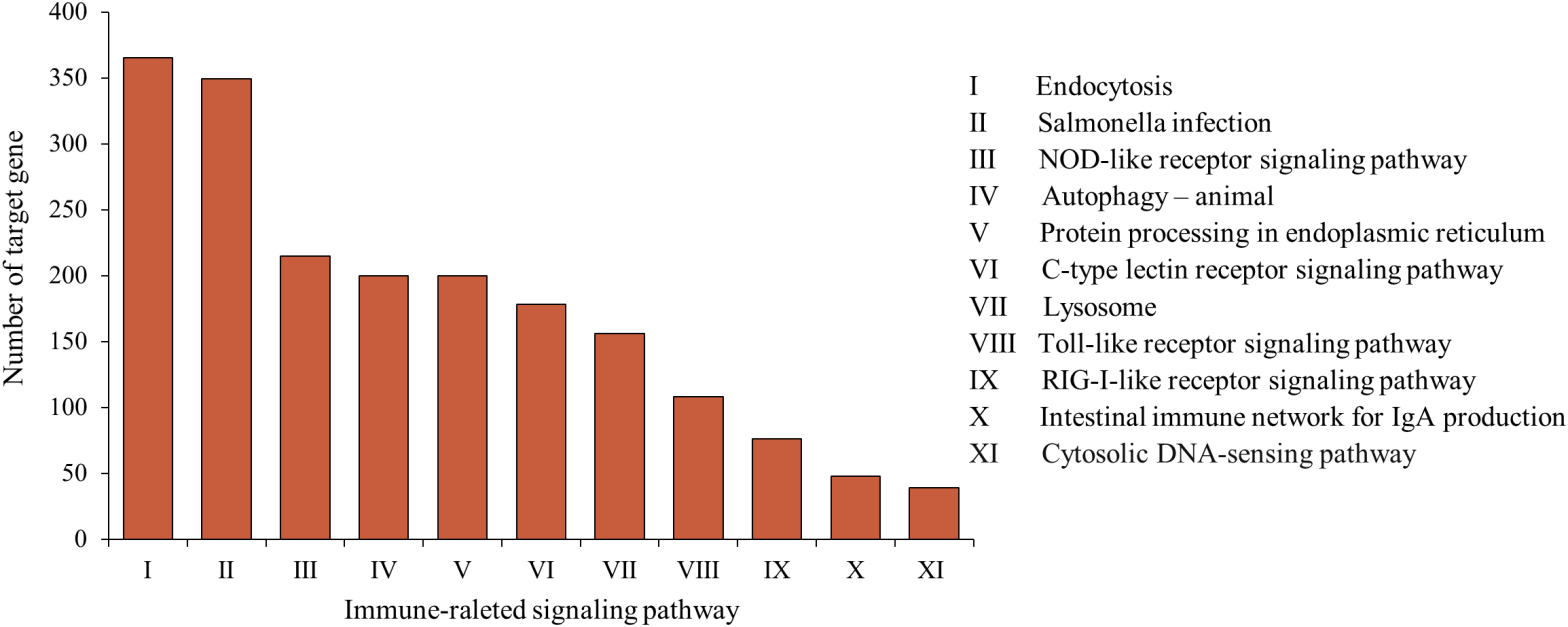
The distribution of genes regulated by these differently expressed miRNAs in immune-related signaling pathway.

A number of studies have reported that fish miRNAs play a crucial role in immunity against pathogens by effectively regulating the immune response. In this study, vine tea extract, as a potential exogenous immunomodulator, induced changes in the expression levels of immune-related miRNAs in loach. KEGG pathway enrichment analysis showed that the target genes of differentially expressed miRNAs were enriched in multiple immune-related signaling pathways (**Figure 7**). Specifically, 200 and 365 target genes were annotated in the autophagy and endocytosis pathways, respectively. These pathways are regulated by the majority of differentially expressed miRNAs and represent fundamental physiological processes by which organisms respond to infectious pathogens, serving as prerequisites for the activation of subsequent immune responses. Moreover, 178, 215, 108, and 76 candidate genes were involved in the C-type lectin receptor signaling pathway, NOD-like receptor signaling pathway, Toll-like receptor signaling pathway, and RIG-I-like receptor signaling pathway, respectively. These pattern recognition receptors enable the host sense bacterial invasion and mediates the innate immune response. Collectively, these results support the hypothesis that vine tea extract pretreatment regulates the response of loach to bacterial infection by modulation of miRNA expression.

## 4 Discussion

Vine tea, a traditional Chinese medicinal plant used as a tea-like beverage in folklore, is rich in a variety of nutrients such as flavonoids, polysaccharides, amino acids and trace elements, etc. Previous studies have shown that vine tea and its extracts contain significant anti-infective bioactivities, including antiviral, anti-bacterial, anti-inflammatory, and immune-regulating activities (43, 44). To the best of our knowledge, there are no reports the application vine tea extracts in aquaculture. In fish, vine tea extract may serve as potential phytobiotics for the prevention and treatment of bacterial infections, thereby reducing mortality and economic losses. Many studies have shown that miRNAs play a critical roles in the immune response to bacterial infections in fish. Therefore, using loach as an experimental animal, we aimed to identify miRNAs and analyze miRNA expression profiles by high-throughput sequencing to understand how vine tea extract modulates immune responses during bacterial infections. In this study, livers from loach before and after treatment with vine tea extract, followed by infection with *A. hydrophila* were collected. Two small RNA libraries were constructed and sequenced using high-throughput deep sequencing technology. A total of 1099 conserved miRNAs were identified by removing non-coding RNAs and repetitive sequences. Differentially expressed miRNAs were then subjected to target gene prediction and functional analysis.

The reads distributions of both libraries indicate that the sequences with the highest abundance are 22 nt in length, which is the typical length of animal miRNAs. This is consistent with the miRNAs length distributions observed in other fish species, such as *Siniperca chuatsi* (45), *Miichthys miiuy* (46), *Ctenopharyngodon Idella* (47), c*ophthalmus maximus* (48), all which show a main peak centered at 22nt centered. Sice the whole genome sequence of loach is not yet available in the miRBase database, the high-throughput sequencing results were compared with mature miRNA sequences from other animal species to identify conserved miRNAs. A total of 1099 miRNAs were finally identified, originating from different species. Analysis of miRNA expression profiles revealed a wide range in reads counts, from as low as 0 to as high as 6,712,291, indicating substantial differences in expression levels among different miRNAs. The most highly expressed miRNA showed similarity to miR-122-5p in rabbits. Rabbit miR-122-5p (ocu-miR-122-5p) has been demonstrated to control gene associated with acute liver failure (ALF) (49). Further investigation is required to determine whether this miRNA also plays a biological role in regulating liver inflammatory response in loach. Since no reference genome was available for alignment and no novel miRNA prediction analyses was performed, some data potentially relevant to the purpose of the study may have been overlooked.

Immunity-associated miRNAs have been identified in several fish model and non-model organisms, where they play diverse roles in the immune response, either promoting the immune response or participate in immune escape mechanisms. In this study, a total of 25 significantly differentially expressed miRNAs were identified when comparing the loach groups treated and untreated with vine tea extract. To verify the reliability of high-throughput sequencing results, RT-qPCR were performed on six miRNAs: manu-mir-194-3, manu-undef-1012, manu-undef-834, manu-undef-882, manu-mir-19-2, and manu-mir-130-16. The qPCR results that were consistent with deep-sequencing analysis. Functional enrichment analysis of the target genes regulated by these differentially expressed miRNAs indicated that multiple miRNAs are involved in signaling pathways associated with the innate immune response. Notably, many of these immune pathways are mediated through the targeting of pattern recognition receptors and downstream signaling factors.

Pattern recognition receptors (PRRs) are a class of biomolecules that recognize pathogen-associated molecular patterns (PAMPs) or damage-associated molecular patterns (DAMPs), and there are four classes of CLRs, NLRs, TLRs and RLRs (50). When PRR is activated by PAMP or DAMP, it transduces signals to the intracellular compartment and activates transcription factors localized in the cytoplasm, which enter the nucleus and activate the transcription of relevant genes, thereby releasing cytokines, interferons or other signaling molecules to rapidly mediate the natural immune response (51). In the study of immune regulation by fish miRNAs, there are many reports of PRRs-mediated immune responses. For instance, Xu et al. found that some genes in the TLR signaling pathway are negatively regulated by differentially expressed miRNAs, which facilitates the activation of transcription factors such as AP - 1, IRF5, NF - kB, and IRF3, and thus enhances host immune responses to clear infectious pathogens (46). In addition, Sun et al. reported that, under poly(I:C) stimulation, miiuy croaker miR-210 could participate in the regulation of the RLR signaling pathway by targeting the DUBA, which could protect the host from viral infection (18). In this research, the functions of the target genes of differentially expressed miRNAs were mainly involved in innate immune-related pathways such as the RIG-I signaling pathway and the TLR signaling pathway, which is similar to previous studies. Interestingly, Xiao et al. demonstrated that vine tea extract shows strong antibacterial activity, with its mechanism potentially related to suppression of the tricarboxylic acid cycle pathway (51). Our finding that the tricarboxylic acid cycle pathway is indeed significantly regulated by differentially expressed miRNAs implies that miRNAs also mediate other non-immune pathways that regulate antibacterial effects.

Beyond this, 25 differentially expressed miRNAs in response to vine tea administration, many of which are closely related to immune regulation. While most of these miRNAs remain uncharacterized, some have been previously demonstrated in other animal species, offering valuable insights into their potential biological roles. For examples, miR-194-3p has been described to function as a tumor suppressor through targeting genes involved in migration and cell proliferation, including *MMP9*. In addition to its anti-tumor role, it also governs immune responses via suppressing the NF-κB pathway and impacts autophagy via targeting *SIRT1*, thereby influencing viral replication (52). Likewise, miR-19, a member of the well-studied miR-17–92 cluster, plays a dual biological role in boosting cell proliferation and controlling immune responses and apoptosis (53). Extending this functional diversity, miR-31 has been implicated in the modulation of integrin expression, which influence migration and cell adhesion; notably, it can function either as an oncogene or a tumor suppressor depending on the cellular context (54). In the context of host-pathogen interactions, miR-130-3p has been demonstrated to inhibit antibacterial immune responses in teleost fish through targeting *NOD1*, thereby facilitating bacterial invasion (55). Complementing this, miR-203 also participates to immune regulation in fish via targeting *IRAK4*, which leads to the attenuation of inflammatory responses against Gram-negative bacterial infections (56). Overall, these results suggest that the differentially expressed miRNAs found in response to vine tea may play multifaceted biological roles in immune modulation, with potential implications for host defense and disease susceptibility.

Although this study offers new insights into the immunomodulatory impacts of vine tea supplementation and found many differentially expressed miRNAs, there are some limitations that should be acknowledged. First, the biological roles of the miRNAs identified in this study were inferred by literature comparisons and bioinformatic predictions; experimental confirmation, including gene expression analysis, miRNA inhibitor/mimic experiments studies, is essential to verify their direct contribution in immune modulation. Second, this study determined the impacts of vine tea supplementation over a short experimental period. The long-term effects of vine tea on health of fish, robustness of immunity, and resistance to diseases remain largely unknown and warrant further studies. Future studies should focus on confirming the crucial miRNA and mRNA interactions, evaluating the dose-dependent influences of vine tea, and performing extended feeding trials to assess the sustainability of its application in aquaculture.

Taken together, in terms of mitigating the threat of bacterial infectious diseases to the loach farming industry, we hope to find a cheap, accessible, effective and environmentally friendly way to do so. We therefore analyzed the expression profiles of bacterial infected loaches with vine tea extract treatment and identified 25 significantly differentially expressed miRNAs. Moreover, the reliability of these miRNA expression profiles was verified by RT - q PCR. Functional annotation of the predicted target genes of differentially expressed miRNAs revealed that the functions of these targets were mainly associated with immune response and antibacterial response. But, to fully elaborate the biological roles of these miRNAs, future studies should focus on experimental confirmation by gene expression analysis, interaction of miRNA-target research, and functional analyses by miRNA inhibitors or mimics. In addition, long-term feeding trials are required to determine the application, optimal dosage, and economic viability of vine tea supplementation in aquaculture operations. It is the first time on the response of miRNA in loach to vine tea extract. Our study deepens our understanding of the function of miRNAs in loach as same as provides a new direction for investigating the application of the antibacterial and immunomodulatory effects of vine tea.

## Data Availability

The datasets presented in this study can be found in online repositories. The names of the repository/repositories and accession number(s) can be found below: https://www.ncbi.nlm.nih.gov/genbank/, SRR29790697.
