# Supplementary material for: Alleviating effect of vine tea on Aeromonas hydrophila infection revealed by small RNA transcriptome analyses of loach liver
Source: Front Immunol. 2025 May 21;16:1584985. doi: 10.3389/fimmu.2025.1584985 (PMC12133533; doi:10.3389/fimmu.2025.1584985)
Supplement: Supplementary file 1 [file Table1.docx]

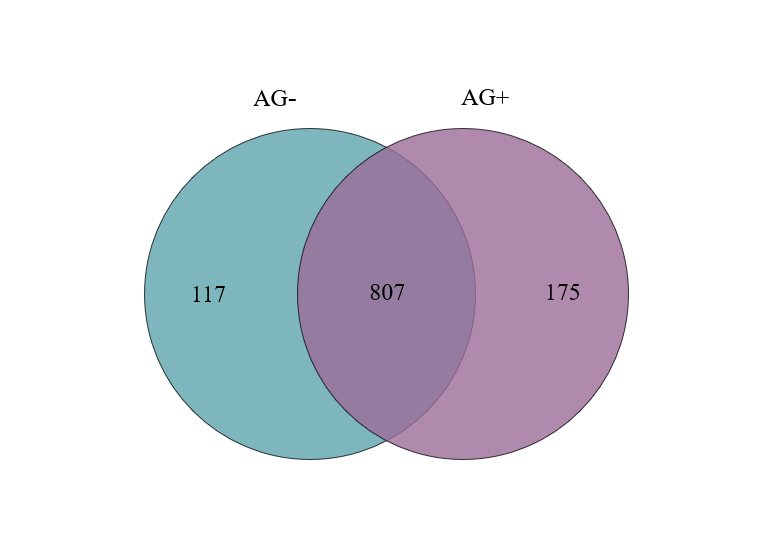


**Additional file 1:** Target prediction by miRanda

|  | **miRNA** | **baseMeanAG-** | **baseMeanAG+** | **baseMean** |
| --- | --- | --- | --- | --- |
| 1 | manu-undef-553 | 6712291.383 | 6588515.549 | 6650403.466 |
| 2 | manu-mir-122-3 | 1750428.332 | 1456522.31 | 1603475.321 |
| 3 | manu-undef-668 | 462440.3111 | 690289.7206 | 576365.0159 |
| 4 | manu-undef-296 | 342297.7892 | 393244.3621 | 367771.0756 |
| 5 | manu-undef-328 | 232106.2472 | 367083.4288 | 299594.838 |
| 6 | manu-undef-327 | 178169.0837 | 291328.7034 | 234748.8936 |
| 7 | manu-mir-148-3 | 161207.8646 | 241676.1167 | 201441.9906 |
| 8 | manu-undef-730 | 193780.4366 | 179512.9198 | 186646.6782 |
| 9 | manu-undef-741 | 155127.3858 | 162086.1671 | 158606.7764 |
| 10 | manu-mir-148-6 | 117341.0795 | 161070.3754 | 139205.7275 |

**Additional file 2:** Top 10 expression abundances in miRNA libraries

|  | **PathwayID** | **Pathway** | **List number** |
| --- | --- | --- | --- |
| 1 | manu04010 | MAPK signaling pathway | 378 |
| 2 | manu04144 | Endocytosis | 365 |
| 3 | manu05132 | Salmonella infection | 349 |
| 4 | manu04810 | Regulation of actin cytoskeleton | 330 |
| 5 | manu04510 | Focal adhesion | 314 |
| 6 | manu05168 | Herpes simplex virus 1 infection | 287 |
| 7 | manu04020 | Calcium signaling pathway | 276 |
| 8 | manu04080 | Neuroactive ligand-receptor interaction | 274 |
| 9 | manu04218 | Cellular senescence | 235 |
| 10 | manu04530 | Tight junction | 234 |

**Additional file 3:** Top 10 pathways with the highest number of target genes involved
